# Identification and validation of COX-2 as a co-target for overcoming cetuximab resistance in colorectal cancer cells

**DOI:** 10.18632/oncotarget.8649

**Published:** 2016-04-08

**Authors:** Yang Lu, Chunmei Shi, Songbo Qiu, Zhen Fan

**Affiliations:** ^1^ Department of Experimental Therapeutics, The University of Texas MD Anderson Cancer Center, Houston, TX 77030, USA; ^2^ Department of Oncology, Fujian Medical University Union Hospital, Fuzhou, Fujian 350001, China

**Keywords:** EGFR, cetuximab, COX-2, COX-2 inhibitor, colorectal cancer

## Abstract

Cetuximab, an epidermal growth factor receptor (EGFR)-blocking antibody, was approved for treatment of metastatic colorectal cancer over a decade ago; however, patients' responses to cetuximab vary substantially due to intrinsic and acquired resistance to cetuximab. Here, we report our findings using Affymetrix HG-U133A array to examine changes in global gene expression between DiFi, a human colorectal cancer cell line that is highly sensitive to cetuximab, and two other cell lines: DiFi5, a DiFi subline with acquired resistance to cetuximab, and DiFi-AG, a DiFi subline with acquired resistance to the EGFR tyrosine kinase inhibitor AG1478 but sensitivity to cetuximab. We identified prostaglandin-endoperoxide synthase 2 (*PTGS2*), which encodes cyclooxygenase-2 (COX-2), as the gene with the greatest difference between the cetuximab-resistant DiFi5 cells and the cetuximab-sensitive DiFi cells and DiFi-AG cells. Reverse transcription polymerase chain reaction and Western blotting validated upregulation of COX-2 in DiFi5 but not in DiFi or DiFi-AG cells. We developed COX-2 knockdown stable clones from DiFi5 cells and demonstrated that genetic knockdown of COX-2 partially re-sensitized DiFi5 cells to cetuximab. We further confirmed that cetuximab in combination with a COX-2 inhibitor led to cell death via apoptosis or autophagy not only in DiFi5 cells but also in another colorectal cancer cell line naturally resistant to cetuximab. Our findings support further evaluation of the strategy of combining cetuximab and a COX-2 inhibitor for treatment of metastatic colorectal cancer.

## INTRODUCTION

Epidermal growth factor receptor (EGFR) is commonly overexpressed in many types of solid tumors. Cetuximab, a mouse-human chimeric antibody that binds to the extracellular domain of EGFR and blocks ligand-induced activation of EGFR [1], has been approved by the US Food and Drug Administration (FDA) for treatment of metastatic colorectal cancer and metastatic head and neck cancer. However, results with cetuximab for these cancers are modest. The year 2004 approval of cetuximab for colorectal cancer under the FDA's accelerated approval program was based largely on the findings from a randomized clinical trial in which cetuximab monotherapy was compared with the combination of cetuximab and irinotecan in irinotecan-refractory metastatic colorectal cancer patients [2]. Results of the trial showed that cetuximab plus irinotecan shrank tumors in 22.9% of patients and was associated with a median time to progression of 4.1 months, whereas cetuximab alone shrank tumors in 10.8% of patients and was associated with a median time to progression of 1.5 months [2]. Although cetuximab shrank tumors in some patients and delayed tumor growth, especially when used in combination with irinotecan, the treatment did not increase overall survival. Later studies have shown that tumors with *RAS* mutation, which is common in colorectal cancer patients, do not respond well to cetuximab [3–9]. Current National Comprehensive Cancer Network guidelines (2014) include the requirement of genotyping for *RAS* mutations (K-*RAS* and N-*RAS*) before cetuximab treatment in patients with metastatic colorectal cancer. According to the guidelines, cetuximab is not recommended for first-line single-agent treatment for metastatic colorectal cancer unless the patient is unable to tolerate irinotecan.

Considerable efforts are being made to improve the efficacy of cetuximab against colorectal cancer through rational, mechanism-based development of combinations of cetuximab and other agents, and one combination that has been tested is dual blockade of the EGFR and cyclooxygenase-2 (COX-2) pathways [10], which play complementary roles in the pathogenesis of cancer [11, 12]. COX-2 is overexpressed in many premalignant and malignant tissues, particularly in organs of the gastrointestinal tract [13, 14]. Epidemiologic studies have shown that nonsteroidal anti-inflammatory drugs (NSAIDs), such as aspirin, significantly reduce the risk of adenomatous polyps or colorectal cancer, and COX-2 is believed to be the prime target of NSAIDs' action [15]. Treatment with a selective COX-2 inhibitor, celecoxib, has shown promising results in colorectal cancer prevention [16, 17]. In the laboratory, cross-mating of COX-2 knockout mice with MIN mice, which have mutation in the adenomatous polyposis coli (*APC*) gene, resulted in mice with significantly reduced formation of intestinal adenomas and cancers associated with *APC* mutation [18]. Moreover, an early clinical study showed that among patients treated with cetuximab, patients with lower expression of COX-2 had a significantly higher rate of grade 2 to 3 skin reactions, which were a biomarker of response after cetuximab treatment [19]. A case report showed a partial response of colorectal cancer to the combination of cetuximab and celecoxib [20]. Furthermore, analysis of tissue samples from 130 participants in the IMC-0144 trial of cetuximab in patients with metastatic colorectal cancer showed that polymorphisms in *PTGS2*, which encodes COX-2, and *EGFR* predicted progression-free survival independently of K-*RAS* mutation status [21]. However, a phase II trial to explore the clinical and biological effects of combined blockade of the EGFR and COX-2 pathways using cetuximab and celecoxib was terminated early owing to lack of sufficient clinical activity and lack of laboratory evidence that the drugs were actually blocking EGFR and COX-2 activity [10]. Therefore, whether dual blockade of EGFR and COX-2 pathways represents a rational approach to benefit colorectal cancer patients remains elusive.

Here, we report findings from our study to identify differences in global gene expression between DiFi human colorectal cancer cells; DiFi5, a DiFi subline with acquired resistance to cetuximab; and DiFi-AG, a DiFi subline with acquired resistance to an EGFR tyrosine kinase inhibitor (TKI). Our study independently identified *PTGS2* as the gene with the greatest difference in expression between cetuximab-resistant DiFi5 cells and cetuximab-sensitive DiFi and DiFi-AG cells. We next performed several functional studies using both genetic and pharmacological approaches to validate COX-2 upregulation as a major mechanism conferring resistance to cetuximab. Our results provide important mechanistic data supporting dual targeting of EGFR and COX-2 as a rational approach for treating metastatic colorectal cancer.

## RESULTS

### Characterization of EGFR inhibition-resistant DiFi sublines and identification of genes differentially expressed between cetuximab-sensitive DiFi cells and cetuximab-resistant DiFi subline cells

**Figure 1:**
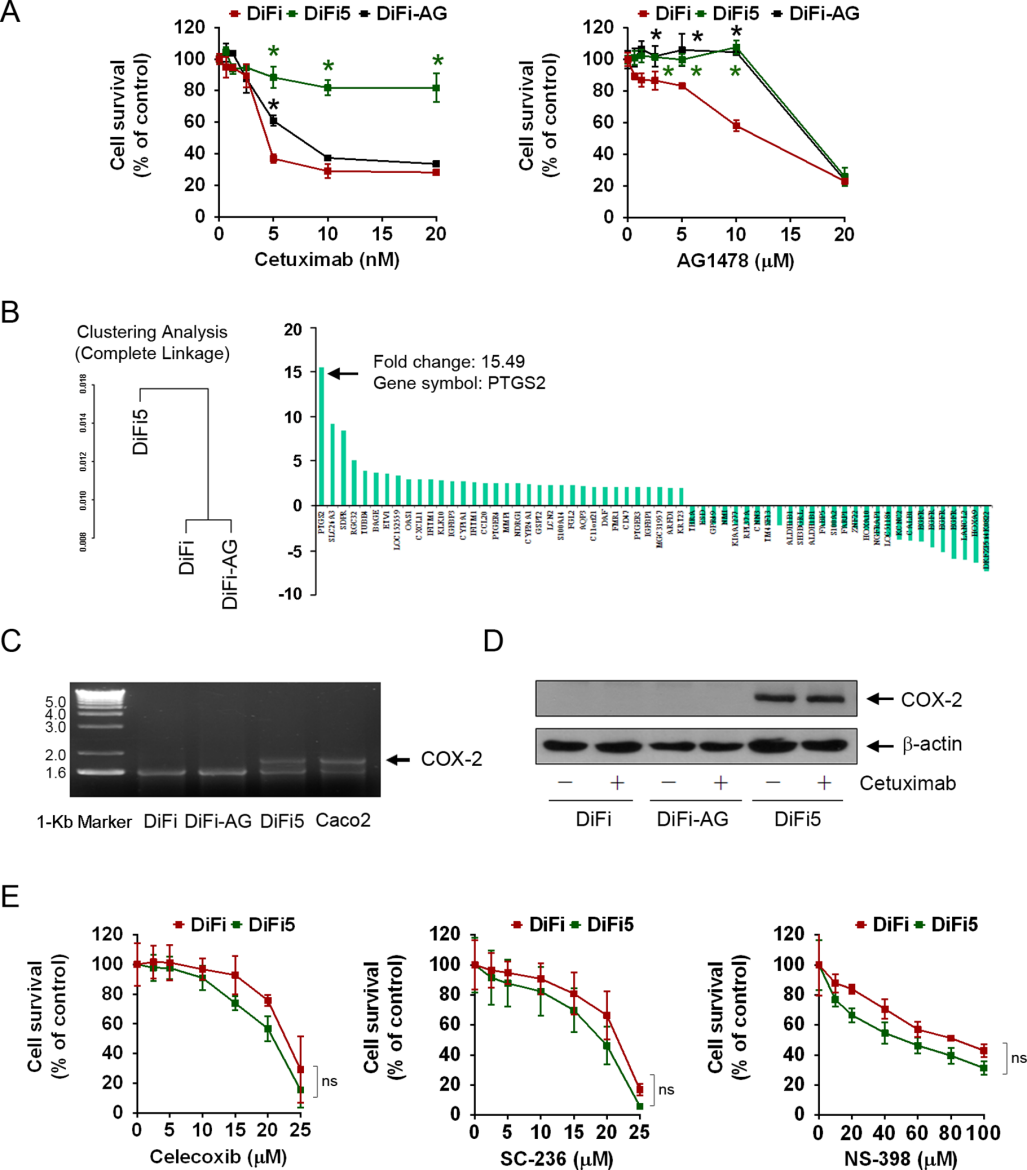
Characterization of EGFR inhibition-resistant DiFi sublines and identification of genes differentially expressed between cetuximab-sensitive and cetuximab-resistant DiFi cells (**A**) DiFi, DiFi5, and DiFi-AG cells were cultured in 0.5% fetal bovine serum containing the indicated concentrations of cetuximab or AG1478 for 5 days and then subjected to MTT assays. The data shown are the optical density (OD) values of treated cell groups at the end of treatment, expressed as a percentage of the OD value of the corresponding untreated or vehicle-treated cells. The color matched *symbols indicate *p* < 0.05 for comparison of the values of DiFi5 or DiFi-AG with corresponding values of DiFi cells. (**B**) Results from Affymetrix HG-U133A microarray gene expression analysis. Complete linkage analysis of gene expression classified DiFi5 cells in a cluster distinct from DiFi and DiFi-AG cells. The waterfall graph shows results from one of two independent analyses for the 62 genes with fold change greater than 2 between the two clusters. *PTGS2* had the highest level of fold change. Additional information is presented in the GEO database (access number GSE71210). (**C**) Total RNA isolated from the indicated cell lines was subjected to RT-PCR amplification using a pair of COX-2-specific primers. The RT-PCR products were analyzed by electrophoresis in a 1.5% agarose gel stained with ethidium bromide and visualized with UV light. (**D**) Cell lysates from the indicated cell lines were subjected to Western blot analysis using a COX-2-specific antibody. The level of β-actin was used as a protein-loading control in each lane. (**E**) DiFi and DiFi5 cells were cultured in 0.5% fetal bovine serum containing the indicated concentrations of celecoxib, SC-236, or NS-398 for 3 days and then subjected to MTT assays as described in (A). ns, not significant for comparison of the values between DiFi and DiFi5 at each dose point for celecoxib, SC-236 and NS-398.

DiFi human colorectal cancer cells exhibit unusually high sensitivity to EGFR inhibition: the cells readily undergo apoptosis after treatment with EGFR-blocking monoclonal antibodies or EGFR TKIs [22–27]. We previously reported generation and characterization of DiFi5, a cetuximab-resistant DiFi subline, through chronic exposure of parental DiFi cells to cetuximab with stepwise increase in concentrations in culture medium [27]. We later adopted a similar approach to generate a DiFi subline with acquired resistance to the EGFR TKI AG1478. This subline, termed DiFi-AG, exhibited strong resistance to AG1478 up to 10 μM (Figure 1A, right panel). However, DiFi-AG cells remained considerably sensitive to cetuximab (Figure 1A, left panel). In contrast, DiFi5 cells are resistant to both cetuximab and AG1478 (Figure 1A). This interesting finding indicates that different mechanisms underlie development of resistance to EGFR inhibitors with different mechanisms of action (i.e., cetuximab versus AG1478). The differences between DiFi5 and DiFi-AG cells in response to cetuximab and AG1478 suggested that these cell lines could be used to identify pathways uniquely associated with response to cetuximab.

We next conducted gene expression profiling of DiFi, DiFi5, and DiFi-AG cells. Results of cluster analysis by the complete-linkage clustering method are shown in Figure 1B, which clustered DiFi-AG cells with DiFi cells and classified DiFi5 cells in a separate cluster. The complete-linkage clustering analysis showed generally similar gene expression profiles in the parental DiFi and DiFi-AG cells, in agreement with our finding that they had similar sensitivity to cetuximab (Figure 1A). We identified 1309 genes with at least a 1.2-fold difference in expression between the cetuximab-resistant DiFi5 cells and the two cetuximab-sensitive cell lines. Detailed information on the 1309 genes (802 genes upregulated and 507 genes downregulated) is presented in the GEO database (access number GSE71210). The 62 genes with fold change greater than 2 are listed in Figure 1B, in order from the highest positive fold change to the highest negative fold change.

**Figure 2:**
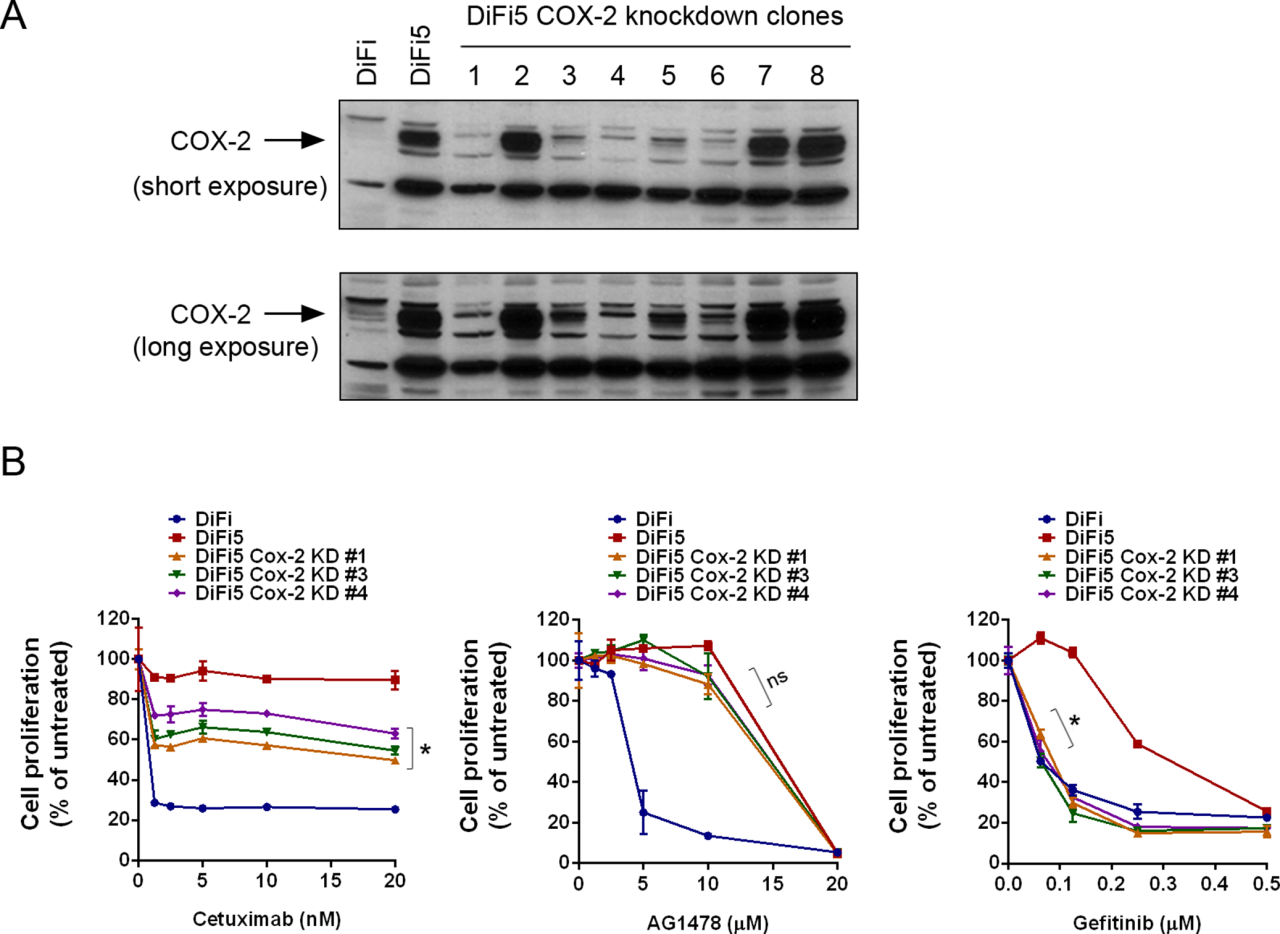
Knockdown of COX-2 differentially resensitizes DiF5 cells to treatment with cetuximab, AG1478, or gefitinib (**A**) DiFi5 cells were transfected with a COX-2 shRNA. Stable clones were selected and analyzed by Western blot analysis, along with parental DiFi and control vector-transfected DiFi5 cells, using a COX-2-specific antibody. Both short and long exposures of the results are shown. (**B**) DiFi, DiFi5, and three COX-2 knockdown DiFi5 clones were cultured in 0.5% fetal bovine serum medium containing indicated concentrations of cetuximab, AG1478, or gefitinib for 3 days and then subjected to an MTT assay. The data shown are the optical density (OD) values of untreated and treated cell groups at the end of treatment, expressed as a percentage of the OD value of corresponding untreated or vehicle-treated cells. *p < 0.05 for comparison of the values of DiFi5 COX-2 knockdown clones with the values of DiFi5; ns, not significant.

*PTGS2*, which encodes COX-2, had the highest positive fold change value in two independent microarray analyses, which showed fold changes values of 15.49 and 10.33, respectively. To validate this finding, we isolated mRNA from DiFi, DiFi5, and DiFi-AG cells and performed RT-PCR using a pair of COX-2-specific primers (Figure 1C). A colorectal cancer cell line, Caco2, which is known to overexpress COX-2 [28] was used as a positive control. The RT-PCR results confirmed that the transcription level of COX-2 was indeed higher in DiFi5 cells than in DiFi and DiFi-AG cells. Further validating our finding, Western blot analysis using a COX-2-specific antibody showed a high level of COX-2 protein in DiFi5 cells, compared to a barely detectable level of COX-2 protein in parental DiFi and DiFi-AG cells in the experiment (Figure 1D); however, DiFi cells did express COX-2 that could be detected after extended exposure of an X-ray film (*see* an overexposed Cox-2 Western blot in Figure 2A).

To determine whether the increase in the level of COX-2 in DiFi5 cells plays a functional role in cell survival, we compared dose-dependent responses of DiFi and DiFi5 cells to treatment with celecoxib, a clinically approved COX-2-specific inhibitor, as well as to treatment with SC-236 and NS-398, two other COX-2 inhibitors commonly used according to reports in the literature. Interestingly, we found no significant difference in sensitivity to COX-2 inhibition-induced cytotoxic effects between DiFi and DiFi5 cells (Figure 1E). This observation suggests that the high level of COX-2 in DiFi5 cells does not render them more dependent than DiFi cells on COX-2 for survival.

### Knockdown of COX-2 partially resensitizes DiFi5 cells to cetuximab via induction of apoptosis but results in differential response to treatment with AG1478 and gefitinib

Next, to determine whether COX-2 plays a role in conferring resistance of DiFi5 cells to cetuximab, we used RNA interference to establish several COX-2 knockdown DiFi5 clones and investigated whether COX-2 knockdown could re-sensitize DiFi5 cells to cetuximab. As shown in Figure 2A, in five of eight selected DiFi5 clones after COX-2 knockdown, the level of COX-2 protein was reduced to a level similar to the low level of COX-2 protein in parental DiFi cells. Three of these five clones were successfully expanded, and their responses to cetuximab were evaluated (Figure 2B). All three clones were significantly more sensitive to cetuximab-induced inhibition of cell proliferation than were DiFi5 cells; however, none of the three COX-2 knockdown clones was as sensitive to cetuximab as were the parental DiFi cells (Figure 2B). This finding indicates that upregulation of COX-2 expression does play an important role in conferring acquired resistance to cetuximab, but other targets may also contribute to the acquired resistance to cetuximab in DiFi5 cells.

It was interesting that the DiFi5 COX-2 knockdown clones remained resistant to the EGFR TKI AG1478, similar to DiFi5 cells, but became sensitive to gefitinib, an FDA-approved EGFR TKI that is highly specific for EGFR (Figure 2B). Compared to gefitinib, AG1478, which was developed earlier, is less specific for EGFR and also a less potent inhibitor of EGFR. In contrast with gefitinib and AG1478, cetuximab inhibits EGFR through blocking ligand-induced activation of receptor tyrosine kinase and through inducing receptor internalization upon binding to EGFR. These observations suggest that although cetuximab, AG1478, and gefitinib all target EGFR, there are distinctions between them in terms of specificity for EGFR, efficacy, and potency to induce cytotoxicity after inhibiting EGFR.

**Figure 3:**
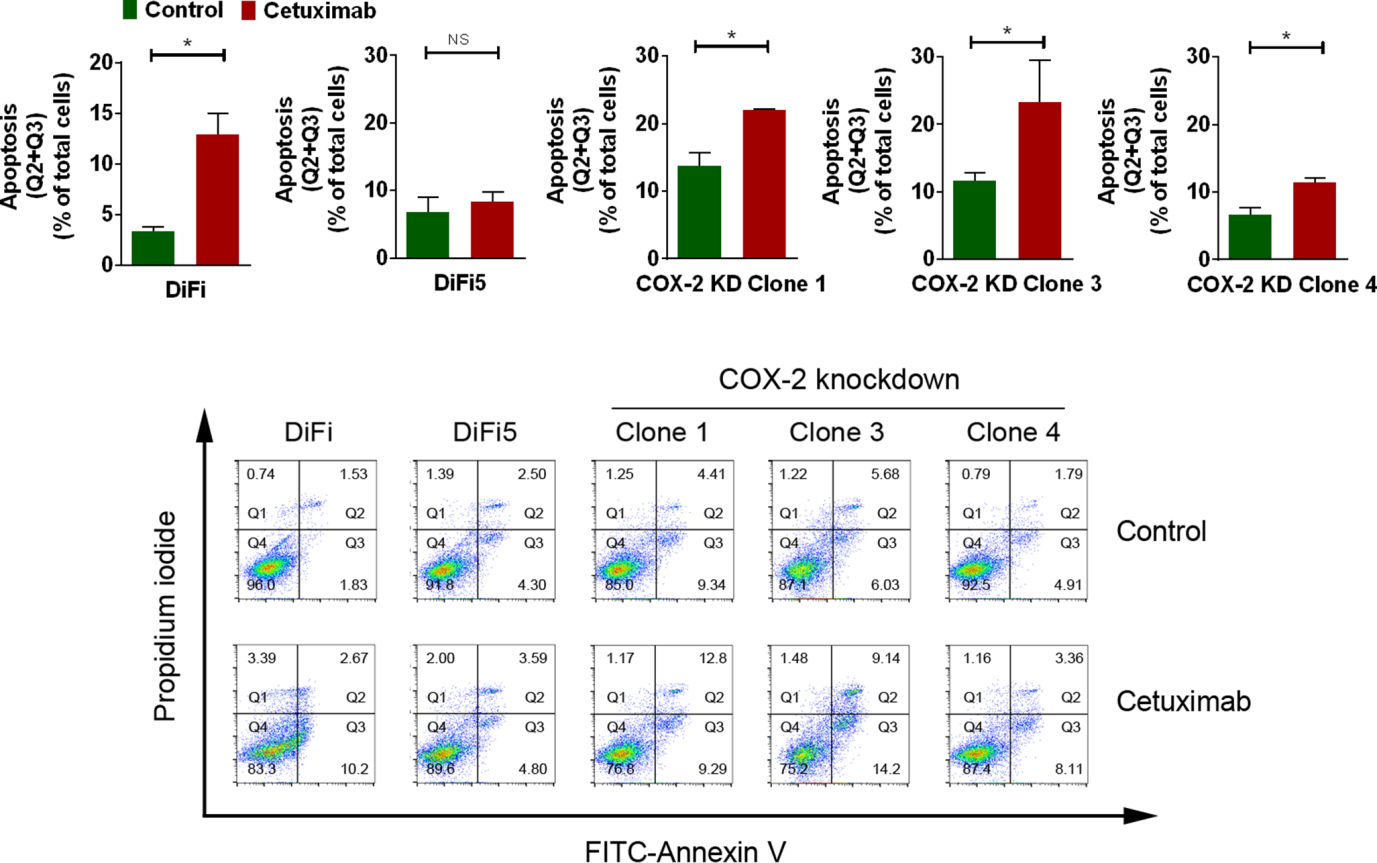
Knockdown of COX-2 partially resensitizes DiFi5 cells to cetuximab via induction of apoptosis DiFi, DiFi5, and DiFi5 COX-2 knockdown clones were treated with 20 nM cetuximab or not for 24 h. Cell samples were subjected to flow cytometry analysis after staining with FITC-labeled Annexin V. Top: Individual comparisons for the sum of Q2 (late apoptotic cells) and Q3 (early apoptotic cells) derived from the contour plots shown at the bottom. KD, knockdown. Unpaired Student *t* test was performed. *p < 0.05; ns, not significant. Bottom: FITC-Annexin V versus propidium iodide contour plots with quadrant gates showing four populations of DiFi, DiFi5, and DiFi5 COX-2 knockdown clones with and without cetuximab treatment.

To focus on the effect of COX-2 knockdown on re-sensitizing DiFi5 cells to cetuximab, which is clinically approved for treating colorectal cancer, we measured the induction of apoptosis by quantifying the percentage of Annexin V-positive cells using flow cytometry analysis. As shown in Figure 3, all three DiFi5 COX-2 knockdown clones were more sensitive to cetuximab treatment than were DiF5 cells, shown by higher percentages of Annexin V-positive cells in the COX-2 knockdown clones than in DiFi5 cells.

Taken together, these data indicate that while the increased COX-2 level in DiFi5 cells does not seem to directly render them more dependent on COX-2 or more sensitive to treatment by COX-2 inhibitors than DiFi cells, the high level of COX-2 does contribute to the acquired resistance of DiFi5 cells to cetuximab-induced apoptosis.

### Pharmacological inhibition of COX-2 enzyme activity resensitizes DiFi5 cells to cetuximab via induction of apoptosis

**Figure 4:**
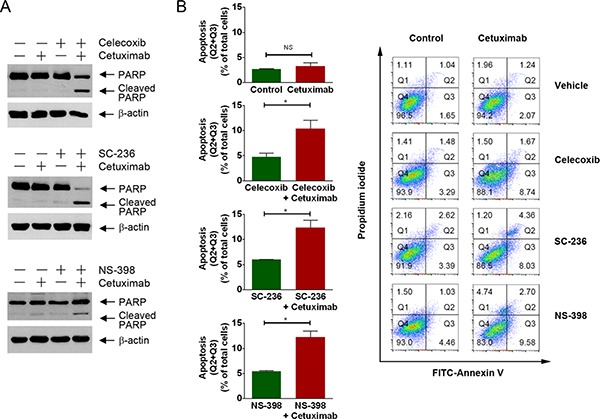
Pharmacological inhibition of COX-2 enzyme activity resensitizes DiFi5 cells to cetuximab via induction of apoptosis (**A** and **B**) DiFi5 cells were treated with cetuximab (20 nM), celecoxib (20 μM), SC-236 (20 μM), and NS-398 (100 μM) alone or in combination as indicated for 24 h. Cell lysates were subjected to Western blotting analysis using the indicated antibodies (A) and subjected to flow cytometry analysis after staining with FITC-labeled Annexin V (B). In B, plots at left show individual comparisons for the sum of Q2 (late apoptotic cells) and Q3 (early apoptotic cells) derived from the contour plots shown at the right. Unpaired Student *t* test was performed. *p < 0.05; ns, not significant. Plots at right are FITC-Annexin V versus propidium iodide contour plots with quadrant gates showing four populations of DiFi5 cells untreated or treated as indicated.

To determine whether inhibition of COX-2 enzyme activity is a promising approach for sensitizing colorectal cancer cells to cetuximab, we examined whether treatment of DiFi5 cells with one of the COX-2 inhibitors used in Figure 1E could achieve an enhanced therapeutic effect through induction of apoptosis in cetuximab-resistant colorectal cancer cells when used in combination with cetuximab. Figure 4 shows that, compared to the results of single treatment of the cetuximab-resistant DiFi5 cells with either cetuximab or any of the COX inhibitors (celecoxib, SC-236, and NS-398), combinations of cetuximab with each of the COX-2 inhibitors induced strong apoptosis in DiFi5 cells, shown by appearance of PARP cleavage detected by Western blotting (Figure 4A) and by increased percentage of Annexin V-positive cells measured by flow cytometry (Figure 4B).

Unlike parental DiFi cells, which are tumorigenic in nude mice, DiFi5 cells grew poorly in nude mice (data not shown). This limitation prevented us from further examining the activity of combinations of cetuximab and a COX-2 inhibitor *in vivo*.

Taken together with the findings from knockdown of COX-2 by RNA interference and inhibition of COX-2 enzyme activity by pharmacological inhibition, our data strongly support the conclusion that upregulation of COX-2 is an important mechanism underlying development of acquired resistance to cetuximab in DiFi5 cells.

### Naturally cetuximab-resistant colorectal cancer cells are sensitive to the combination of cetuximab and COX-2 inhibition

**Figure 5:**
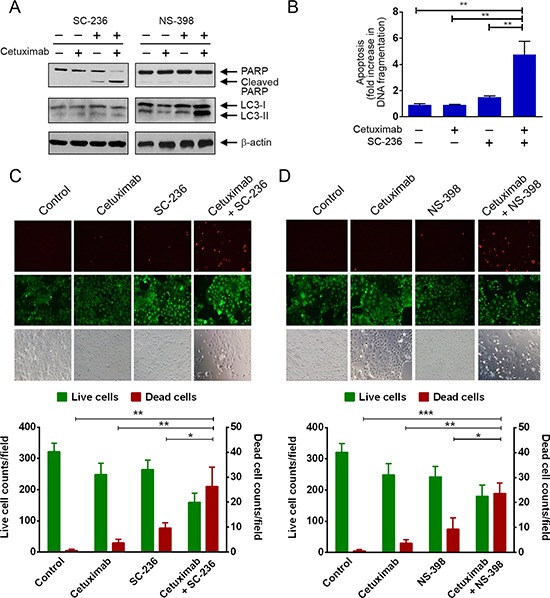
Pharmacological inhibition of COX-2 enzyme activity sensitizes Caco2 colorectal cancer cells to cetuximab via induction of apoptosis or autophagy (**A**) Caco2 cells were treated with cetuximab (20 nM) for 24 h and/or SC-236 (100 μM) for 3 h (left panel) or with cetuximab (20 nM) for 24 h and/or NS-398 (100 μM) for 24 h (right panel) before cell lysis. Cell lysates were subjected to Western blotting analysis using the indicated antibodies. (**B**) Caco2 cells treated with cetuximab and/or SC-236 in (A) were subjected to a quantitative apoptosis ELISA. (**C**) and (**D**) Caco2 cells were treated as described in (A). After the treatments, cells were subjected to a LIVE/DEAD assay and observed under a fluorescent microscope. In, B, C, and D, unpaired Student *t* test was performed. *p < 0.05; **p < 0.01.

To test whether cetuximab plus inhibition of COX-2 is an effective approach for treating COX-2-overexpressing colorectal cancer cells with naturally occurring resistance to cetuximab, we performed a proof-of-concept study to examine the effect of combination treatment with cetuximab and a COX-2 inhibitor in Caco2 cells. Caco2 cells are resistant to cetuximab, shown by lack of induction of apoptosis (Figure 5). However, these cells were sensitive to COX-2 inhibition. Treatment of the cells with SC-236 alone induced PARP cleavage (Figure 5A). Importantly, the level of apoptosis was higher with the combination of SC-236 and cetuximab than with either single treatment alone (Figure 5A, left panel, and Figure 5B). This result was highly similar to our finding with the same combination treatment in DiFi5 cells (Figure 4). Interestingly, when we replaced SC-236 with NS-398, we did not find signs of apoptosis measured by PARP cleavage (Figure 5A, right panel); instead, we detected a higher level of LC3-II, which is indicative of autophagosome formation and the amount of which is correlated with the extent of autophagy [29].

To confirm that cell death was indeed induced after treatment with the combination of cetuximab with SC-236 or NS-398, we used a fluorescence-based live/dead cell assay. We found that although cetuximab plus SC-236 appeared to lead to apoptosis whereas cetuximab plus NS-298 appeared to lead to autophagy, both combination treatments induced significant cell death of Caco2 cells, shown by marked increase in the number of dead cells, identified by bright red fluorescence (Figure 5C and 5D).

In conclusion, our results support the strategy of inhibition of COX-2 as a rational approach to overcoming cetuximab resistance of colorectal cancer cells.

## DISCUSSION

In this study, we searched for global gene expression changes associated with cetuximab resistance in colorectal cancer cells. Among 1309 genes identified to be upregulated or downregulated in the cetuximab-resistant (DiFi5) cells compared with the cetuximab-sensitive (DiFi and DiFi-AG) cells, we found that the gene encoding COX-2, which was upregulated in DiFi5 cells, was the gene with the highest level of fold change in expression. We confirmed that knockdown of COX-2 by RNA interference or pharmacological inhibition of COX-2 enzyme activity re-sensitized to cetuximab treatment not only DiFi5 cells but also Caco2 cells, colorectal cancer cells that overexpress COX-2 and are naturally resistant to cetuximab.

COX-2 is unexpressed or expressed at a very low level under normal conditions in most cells but is elevated during inflammation and in many cancers, particularly in colorectal cancer [30]. Pharmacological inhibitors of COX-2 are available and have shown antitumor activity in preclinical and clinical studies [16, 31–35]. Unfortunately, a recent clinical trial testing the combination of cetuximab and the COX-2 inhibitor celecoxib failed to show clinical benefits [10]. However, the findings from our current study support EGFR and COX-2 dual blockade and indicate the need for additional clinical trials of this combination strategy, probably with improvement in clinical trial design for achieving better pharmacokinetic and pharmacodynamic results from COX-2 inhibition in targeted cancer cells.

It is interesting that the biochemical mechanisms underlying development of acquired resistance to EGFR blockade with an antibody and EGFR tyrosine kinase inhibition with a small molecule inhibitor were somewhat different. In the current study, we focused mainly on the cetuximab-resistant DiFi5 cells because cetuximab is approved for treatment of colorectal cancer whereas TKIs are not. Of note, AG1478 is an early developed EGFR TKI that is not as specific and potent as the TKIs currently approved for treating non-small cell lung cancer patients, such as gefitinib and erlotinib. Whereas AG1478 clearly can inhibit EGFR tyrosine kinase at the doses we used in our study, it may also interact with some other molecules. It would therefore also be interesting to develop gefitinib- or erlotinib-resistant DiFi cells and examine how they respond to cetuximab treatment.

In the current study, we did not explore the mechanism(s) underlying upregulated COX-2 expression in cetuximab-resistant cells. Previous studies in the literature showed positive crosstalk between COX-2 and EGFR signaling pathways, that is, an increase in COX-2 expression led to an increase in EGFR expression and vice versa [11, 12]. The findings from our study, however, do not seem to fit this paradigm. The fact that COX-2 expression was upregulated in DiFi5 but not in DiFi-AG cells suggests that the underlying mechanism is not related to EGFR inhibition only, which further suggests that the upregulation of COX-2 in DiFi5 cells could be related only to cetuximab treatment. Further in-depth investigation to understand the mechanisms underlying this phenomenon are clearly needed.

It is important to note that we identified 1309 genes whose expression was upregulated or downregulated in DiFi5 cells compared to DiFi and DiFi-AG cells. This indicates that COX-2 is likely not the only gene whose expression upregulation is causally related to development of cetuximab resistance. Furthermore, posttranslational modifications not detected at the gene expression level can also play a role in cetuximab resistance. For example, we previously reported increased activation-specific phosphorylation of Src in DiFi5 cells and reported that the combination of cetuximab and a Src kinase inhibitor improved response of DiFi5 cells to cetuximab [27]. DiFi5 cells appeared to acquire cetuximab resistance in part through activation of Src to compensate for a reduced role of EGFR in regulating cell signaling due to high EGFR ubiquitination at basal level in DiFi5 cells [27].

In summary, we identified and validated upregulation of COX-2 at the gene expression profile level as a major mechanism contributing to the development of cetuximab resistance in colorectal cancer cells. Our data provide a new mechanism-based rationale supporting clinical trials testing the strategy of EGFR and COX-2 dual blockade for treating metastatic colorectal cancer.

## MATERIALS AND METHODS

### Materials

Cetuximab (Erbitux) was obtained from the outpatient pharmacy at The University of Texas MD Anderson Cancer Center. The COX-2 inhibitors celecoxib, SC-236 (4-[5-(4-chlorophenyl)-3-(trifluoromethyl)-1H-pyrazol-1-yl]-benzenesulfonamide), and NS-398 (9N-[2-(cyclohexyloxy)-4-nitrophenyl]-methanesulfonamide) were purchased from Cayman Chemical. All other chemicals were purchased from Sigma-Aldrich unless otherwise specified.

### Cell lines and cell proliferation assay

DiFi human colorectal adenocarcinoma cells and a cetuximab-resistant DiFi subline, DiFi5, were described previously [24–27]. DiFi-AG, a DiFi subline with acquired resistance to AG1478, an EGFR TKI, was generated by chronic exposure to serially increased doses of AG1478 (from 0.1 μM to 5 μM) for over 1 year. Caco2 human colorectal adenocarcinoma cells were purchased from American Type Culture Collection. All cell lines were maintained in a mixture of Dulbecco's modified Eagle's medium and Ham's F-12 medium (1:1, v/v) supplemented with 10% fetal bovine serum in a 37°C humidified atmosphere containing 95% air and 5% CO_2_ and were split twice a week.

To measure cell proliferation following treatments of cells in 24-well plates, 10 mg/mL methylthiazolyldiphenyl-tetrazolium bromide (MTT) was added (50 μL in 0.5 mL medium/well), and the cells were incubated for an additional 2 h. The cells were then lysed with a lysis buffer (500 μL/well) containing 20% sodium dodecyl sulfate in dimethyl formamide/H_2_O (1:1, v/v; pH 4.7) at 37°C for at least 6 h. The relative number of surviving cells in each group was determined by measuring the optical density (OD) of the cell lysates at an absorbance wavelength of 570 nm. The OD value of each treatment group was expressed as a percentage of the OD value of the untreated control cells [36].

### Global gene expression analysis

For global gene expression analysis, total RNA from parental DiFi, DiFi5, and DiFi-AG cells was extracted using Trizol Reagent (Life Technologies) and then subjected to clean-up and DNase digestion on an RNeasy spin column (QIAGEN). RNA was quantified using a UV spectrophotometer, and quality was assessed on agarose gel. Each RNA was then converted into double-strand cDNA using a T7 polymerase (New England BioLabs) and purified. Probes were prepared using standard Affymetrix protocols and hybridized to an Affymetrix HG-U133A array according to the manufacturer's instructions. Following hybridization, the array was scanned using a laser confocal scanner, and microarray image data were analyzed using DNA-Chip Analyzer (dChip), version 1.3, by the Sequencing and Microarray Facility at MD Anderson Cancer Center. Two independent microarray analyses were performed. The microarray data reported in this paper have been submitted to the gene expression omnibus (GEO) database at National Center for Biotechnology Information, with the access number GSE71210.

### Validation of COX-2 gene expression by reverse transcription polymerase chain reaction (RT-PCR)

Total RNA from parental DiFi, DiFi5, and DiFi-AG cells extracted as described above was reverse transcribed into cDNA. RT-PCR amplification of COX-2 was performed using the following pair of primers based on the known COX-2 cDNA sequence (NM_000963.3): forward primer flanked by a *Bam*HI site, CGGGATCCGCCACCATGCTCGCCCGCGCCCTG; reverse primer flanked by an *Eco*RI site, CGGA ATTCCTACAGTTCAGTCGAACGTTCT. Following amplification, 5 μL of each PCR product was analyzed by electrophoresis in a 1.5% agarose gel stained with ethidium bromide. The gels were visualized with UV light and photographed.

### Western blotting

Cells were lysed in a lysis buffer containing 50 mM TrisHCl (pH 7.4), 150 mM NaCl, 0.5% Igepal CA-630, 50 mM NaF, 1 mM Na_3_VO_4_, 1 mM phenylmethylsulfonyl fluoride, 25 μg/mL aprotinin, and 25 μg/mL leupeptin and kept on ice for 15 min [36]. The lysates were cleared by centrifugation, and the supernatants were collected. Equal amounts of protein lysate, as determined using the Pierce Coomassie Plus colorimetric protein assay (Thermo Fisher Scientific), were separated by SDS–polyacrylamide gel electrophoresis, blotted onto nitrocellulose, and probed with primary antibodies against COX-2 (Santa Cruz Biotechnology), poly (ADP-ribose) polymerase (PARP), and microtubule-associated protein 1A/1B-light chain 3 (LC3) (Cell Signaling Technology) and against β-actin (Sigma-Aldrich). The signals were visualized using the enhanced chemiluminescence detection kit (GE Healthcare).

### Establishment of COX-2 knockdown stable clones

DiFi5 cells were transfected with one of several 29-mer COX-2 HuSH-29 shRNA constructs or the control vector shRNA pGFP-V-RS (OriGene Technologies) using Lipofectamine 2000 (Life Technologies) according to the manufacturer's protocols. The COX-2 DNA-targeting sequences that we validated to lead to successful knockdown of COX-2 expression are CAGAG TTGGAAGCACTCTATGGTGACATC (catalogue # TG310074C) and CTGGTGCCTGGTCTGATGAT GTATGCCAC (catalogue # TG310074D). After transfection, the cells were selected with puromycin (200 ng/mL) for puromycin-resistant clones over a few weeks. Surviving clones were examined for the level of COX-2 expression by Western blotting and further expanded for functional analysis.

### LIVE/DEAD cell viability assay

The LIVE/DEAD cell viability assay kit (Life Technologies) was used to detect cell death as recently described [37]. In brief, following treatments, cells were incubated with 4 μM calcein acetoxymethyl ester and 2 μM ethidium homodimer-1 together in a 37°C, 5% CO_2_ incubator for 45 min. The cells were then rinsed gently with phosphate-buffered saline and then observed for cell viability under a fluorescence microscope. Live cells were identified by green fluorescence when excited at 485 nm, and dead cells were identified by a bright red fluorescence when excited at 544 nm. Several different areas were then randomly selected and imaged under a fluorescence microscope. The imaging data were analyzed using the ImageJ software program [38–42].

### Apoptosis assays

Apoptosis was measured by several methods we described previously [43, 44]. The first is to quantitatively measure the percentages of apoptotic cells using a flow cytometer after staining of cells with FITC-conjugated Annexin V and propidium iodide (Life Technologies), according to the vendors' protocols. The second is to detect PARP cleavage using Western blotting with an antibody that recognizes both cleaved and uncleaved PARP (Cell Signaling Technology). The third is to quantitatively measure the levels of cytoplasmic histone-associated DNA fragments (mononucleosomes and oligonucleosomes) using a Cell Death Detection ELISA kit (Roche Diagnostics Corp.).

### Statistical analysis

Each experiment was repeated at least three times, and the mean values with standard error of the mean are presented. A two-tailed unpaired Student's *t* test was used to compare two groups of independent samples. *p* < 0.05 was considered statistically significant.
